# Correlation of SARS-CoV-2 RNA and nucleocapsid concentrations in samples used in INSTAND external quality assessment schemes

**DOI:** 10.1186/s13104-023-06497-7

**Published:** 2023-09-11

**Authors:** Esmeralda Valiente, Samreen Falak, Andreas Kummrow, Martin Kammel, Victor M. Corman, Rainer Macdonald, Heinz Zeichhardt

**Affiliations:** 1 Physikalisch-Technische Bundesanstalt, Department 8.3 - Biomedical Optics Abbestr. 2-12, D-10587 Berlin, Germany; 2 INSTAND e.V, Society for Promoting Quality Assurance in Medical Laboratories, Duesseldorf, North Rhine-Westphalia Germany; 3 GBD Gesellschaft fuer Biotechnologische Diagnostik mbH, Berlin, Germany; 4 Institute of Virology, Charité - Universitaetsmedizin Berlin, National Consultant Laboratory for Coronaviruses, German Centre for Infection Research, Berlin, Germany; 5 Labor Berlin - Charité Vivantes GmbH, Berlin, Germany; 6 IQVD GmbH, Institut fuer Qualitaetssicherung in der Virusdiagnostik, Berlin, Germany

**Keywords:** SARS-CoV-2, Nucleocapsid (NCap) antigen, ELISA, RNA, RT-dPCR, Quantification

## Abstract

**Objective:**

In routine clinical laboratories, severe acute respiratory syndrome coronavirus (SARS-CoV-2) infection is determined by reverse-transcription PCR (RT-PCR). In the COVID pandemic, a wide range of antigen detection tests were also in high demand. We investigated the correlation between SARS-CoV-2 NCap antigen and *N* gene concentration by analyzing samples from several INSTAND external quality assessment (EQA) schemes starting in March 2021. The absolute *N* gene concentration was measured using reverse transcriptase digital PCR (RT-dPCR) as reference value. Moreover, the performance of five commercial ELISA tests using an EQA inactivated SARS-CoV-2 sample at different concentrations was assessed on the basis of these reference values.

**Results:**

Quantitative ELISA and RT-dPCR results showed a good correlation between SARS-CoV-2 NCap antigen and RNA concentration, but this correlation varies among SARS-CoV-2 isolates. A direct correlation between SARS-CoV-2 NCap antigen concentration and genome concentration should not be generally assumed.

**Conclusion:**

Further correlation studies between SARS-CoV-2 RNA and NCap antigen concentrations are needed, particularly in clinical samples and for emerging SARS-CoV-2 variants, to support the monitoring and improvement of antigen testing.

## Introduction

Severe acute respiratory syndrome coronavirus 2 (SARS-CoV-2), first reported in December 2019 in Wuhan, China [1], has turned into a global public health problem [2, 3]. This disease has spawned the development of rapid and sensitive diagnostic tests based on nucleic acids and proteins to detect SARS-CoV-2 [4]. Although antigen detection tests are inferior in terms of sensitivity compared to quantitative real-time PCR (RT-qPCR) for the detection of SARS-CoV-2, they do cover the high demand for early diagnosis due to their ease of use and rapid delivery of results [5, 6].

The correlation of SARS-CoV-2 NCap antigen concentration with cycle threshold (Ct) values such as RNA concentration in clinical samples has been investigated [7]. However, RNA concentration can only be determined from Ct values if a calibrator is available and used, which was not the case in this study. In contrast, we compared calibration-free RNA absolute quantification by RT-dPCR with quantitative NCap antigen ELISAs to characterize the correlation of *N* gene and NCap antigen concentrations by analyzing dilution series of samples from the INSTAND External Quality Assessment (EQA) scheme for SARS-CoV-2 antigen detection (Program 410). For this purpose, we analyzed three quantitative ELISA assays for SARS-CoV-2 NCap antigen quantification and, for comparison, two qualitative ELISAs widely used in SARS-CoV-2 diagnostics. We used serial dilutions of one INSTAND EQA SARS-CoV-2 antigen sample from March 2021. Moreover, the quantitative ELISA kit with the best performance in terms of linearity, limit of detection (LOD) and limit of quantification (LOQ) was consequently used for correlation studies and for testing the samples from the cited EQA schemes of March, September and November 2021 and March 2022.

## Materials and methods

### Sample preparation and distribution

The samples derived from EQA schemes were provided by INSTAND e.V (https://www.instand-ev.de/en/instand-eqas/eqa-program/offer/virus-antigen-detection-sars-cov-2-ag/). INSTAND (Society for Promoting Quality Assurance in Medical Laboratories e.V.) has been designated as a German reference institution for quality assurance in medical laboratories by the German Medical Association and is accredited according to DIN EN ISO/IEC 17043:2010. The samples investigated in this study are shown in Table 1. For the SARS-CoV-2 isolates, aliquots of the corresponding Vero E6 (ATCC CRL-1586) cell culture supernatants were treated with 0.05% BPL for 14 h at 4 °C. BPL was then hydrolyzed at 37 C for two hours. Infectivity (measured in plaque forming units, PFU) was determined by plaque assay. Finally, 0.5 ml of the materials were aliquoted in screw cap micro tubes (2,0 mL, Sarstedt, Nümbrecht, Germany) and lyophilized. Each lyophilized vial was reconstituted in 0.5 ml of molecular water. Randomly selected vials of each of the EQA samples were analyzed for stability during the period of the EQA survey and for homogeneity according to DIN EN ISO/IEC 17043:2010 [8]. Prior to the EQA survey, the EQA samples were tested by 2–5 INSTAND expert laboratories for suitability and declared qualified regarding the specified properties. For the evaluation of the commercial ELISA kits, EQA sample no. 410,004 was used (Table 1). For RNA and protein concentration correlation studies, we examined different dilutions of three SARS-CoV-2 isolates (Table 1; Fig. 2B C). Our participation in the INSTAND EQA Scheme involved the analysis of the sample sets corresponding to Program 410, distributed in September and November 2021 and March 2022 (Table 1).

**Table 1:** Overview of the antigen samples distributed during Program 410 of the INSTAND EQA schemes between 2021 and 2022

INSTAND EQA Scheme	Sample Number	Sample Properties				Quantitative Result	
		SARS-CoV-2 isolate / human coronavirus isolate / negative control cells	Dilution factor	Target value in EQA scheme	Matrix	*N* gene concentration by dPCR (copies/ml)	Antigen mass concentration by Abcam ELISA (pg/ml)
March 2021	410,001	SARS-CoV-2 Non-VOC, isolate: BetaCoV/Munich/ChVir984/2020_IsolatBER	1 : 7 500	not evaluated	cell culture supernatant	1193536.87	106.12
March 2021	410,002	negative control cells (MRS-5)	-	negative	cell lysate	ND	0
March 2021	410,003	human coronavirus (hCoV) 229E	1 : 1 000	negative	cell culture supernatant	ND	0
March 2021	410,004	SARS-CoV-2 Non-VOC, isolate: BetaCoV/Munich/ChVir984/2020_IsolatBER	1 : 750	positive	cell culture supernatant	12097636.53	2509.52
March 2021	410,005	SARS-CoV-2 Non-VOC, isolate: BetaCoV/Munich/ChVir984/2020_IsolatBER	1 : 75 000	not evaluated	cell culture supernatant	91586.48	36.33
September 2021	410,011	negative control cells (MRS-5)	-	negative	cell lysate	ND	0
September 2021	410,012	SARS-CoV-2 Non-VOC, isolate: BetaCoV/Munich/ChVir984/2020_IsolatBER	1 : 750	positive	cell culture supernatant	ND	2577.14
September 2021	410,013	SARS-CoV-2 Non-VOC, isolate: BetaCoV/Munich/ChVir984/2020_IsolatBER	1 :2 371.7	positive	cell culture supernatant	ND	605.54
September 2021	410,014	SARS-CoV-2 Non-VOC, isolate: BetaCoV/Munich/ChVir984/2020_IsolatBER	1 : 7 500	not evaluated	cell culture supernatant	ND	174.23
September 2021	410,015	SARS-CoV-2 Delta VOC, B.1.617.2, isolate: hCoV-19/Germany/SH-CHVir25702_4/2021	1 : 500	positive	cell culture supernatant	13,943,225	615.26
November 2021	410,016	SARS-CoV-2 Non-VOC, isolate: BetaCoV/Munich/ChVir984/2020_IsolatBER	1 : 750	positive	cell culture supernatant	ND	2202.92
November 2021	410,017	SARS-CoV-2 Non-VOC, isolate: BetaCoV/Munich/ChVir984/2020_IsolatBER	1 : 7 500	not evaluated	cell culture supernatant	ND	171.85
November 2021	410,018	negative control cells (MRS-5)	-	negative	cell lysate	ND	0
November 2021	410,019	SARS-CoV-2 Delta VOC, B.1.617.2, isolate: hCoV-19/Germany/SH-CHVir25702_4/2021	1 : 250	positive	cell culture supernatant	61,829,936	1114.32
November 2021	410,020	SARS-CoV-2 Non-VOC, isolate: BetaCoV/Munich/ChVir984/2020_IsolatBER	1 : 2 371.7	positive	cell culture supernatant	ND	589
March 2022	410,021	SARS-CoV-2 Delta VOC, B.1.617.2, isolate: hCoV-19/Germany/SH-CHVir25702_4/2021	1 : 250	positive	cell culture supernatant	70185043.55	1481.05
March 2022	410,022	negative control cells (MRS-5)	-	negative	cell lysate	17.45	5.86
March 2022	410,023	SARS-CoV-2 Omicron VOC BA.1, isolate: hCoV-19/Germany/SH-ChVir26373/2021	1 : 750	positive	cell culture supernatant	6,296,372	220.75
March 2022	410,024	SARS-CoV-2 Non-VOC, isolate: BetaCoV/Munich/ChVir984/2020_IsolatBER	1 : 750	positive	cell culture supernatant	14,165,940	2189.70
March 2022	410,025	SARS-CoV-2 Omicron VOC BA.1, isolate: hCoV-19/Germany/SH-ChVir26373/2021	1 : 250	positive	cell culture supernatant	21,777,778	569.60

### ELISA

The Roche Elecsys SARS-CoV-2 (product number: 09 345 272 190) and the Euroimmun SARS-CoV-2 (product number Eqs. 2606–9601) antigen tests were performed for the qualitative detection of SARS-CoV-2 NCap. The ProteinTech (KE30007), Abcam (Ab274341), and GeneTex (GTX535824) tests were used for quantitative measurements of the SARS-CoV-2 NCap. To compare the dilution linearity of the three quantitative ELISA tests, a highly concentrated SARS-CoV-2 sample (ID 410,004) and a series of five dilutions (2x, 4x, 8x,16x,32x) were examined in duplicate on three different days. For the qualitative ELISAs, just five dilutions (2x, 4x, 8x, 16x, 32x) were examined. Linear regression analysis was performed using Gen5 analysis software. LODs were determined as the concentration of recombinant nucleocapsid that provides a signal at least three times the standard deviation above the assay background generated using the correspondent kit assay diluent as a blank sample. For the LOQ, ten standard deviations were added to the background signal. For the background measurements of the different ELISA kits, 20 replicates were conducted.

### RT-dPCR assay

After RNA extraction with the Qiagen QiaAmp RNA kit, one step RT-dPCR reactions were carried out as described in [9]. For *N* gene quantification, a duplex assay was performed using *China N* and *SarE* genes with primers and probe concentrations of 400 nM and 200 nM, respectively, and optimized by varying the annealing temperature. The sequence of China *N* and *SarE* primers is described in [9–11], Droplet generation was conducted as described in [9]. The PCR was performed under the following conditions: 60 min reverse transcription at 50 ºC and 10 min enzyme inactivation at 95 ºC followed by 45 cycles using a two-step thermal profile of 30 s denaturation at 95 ºC and 60 s annealing and extension at 55 ºC; followed by 10 min at 98 ºC and then cooled to 4 ºC. Following thermal cycling, the PCR plates were transferred to a droplet reader (QX200 BioRad, USA) and the data analyzed using QuantaSoft Analysis Pro 1.0.596 (BioRad, USA).

### EQA participation

We participated in the Virus Antigen Detection SARS-CoV-2 EQA scheme (Program 410) of INSTAND in September/November 2021 and March 2022 together with 200 laboratories and used the Abcam ELISA kit for NCap antigen testing. Each program covers chemically inactivated samples from three different sources (Table 1). EQA Program 410 requires qualitative results from each EQA sample.

### Statistical analysis

Statistical analyses were performed using Origin 2019 software (OriginLab Corporation, USA). The results of data analysis are presented as descriptive statistics by mean and standard deviation as appropriate. As a non-normal distribution was confirmed by a Kolmogorov–Smirnov test, a non-parametric Mann–Whitney U test was used to compare the SARS-CoV-2 nucleocapsid concentration between the ELISA kits. For all statistical analyses, *p*-values < 0.05 were deemed statistically significant.

## Results

**Fig. 1:**
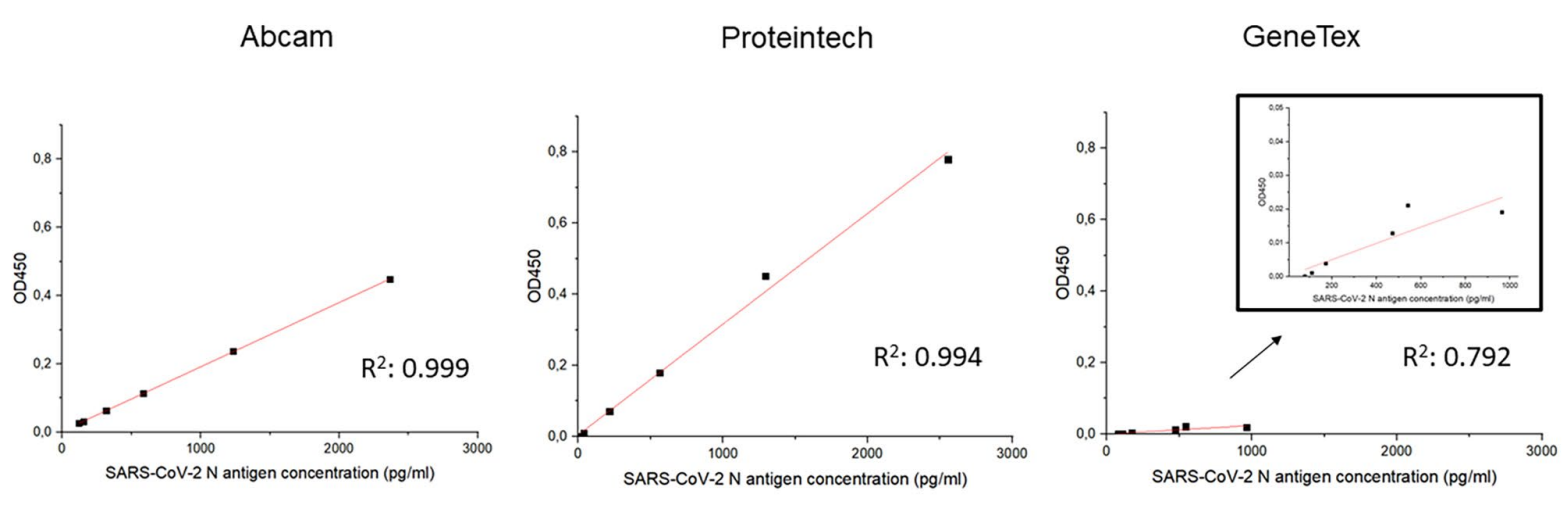
Dilution linearity comparison of the nucleocapsid concentration of the three quantitative commercial ELISA kits (Abcam, ab274341; ProteinTech, KE30007; GeneTex, GTX535824) using serial dilutions (2x, 4x, 8x,16x and 32x) of EQA SARS-CoV-2 sample (ID 410,004, Table 1**).** Mean optical density (OD) values were obtained according to the kit’s specifications and the measurements were obtained using a BioTek Synergy H4 plate reader (Biotek GmbH). Linear regression analysis was performed using Gen5 analysis software. The LOD and LOQ of the different kits were 79 pg/ml and 130 pg/ml, respectively, for the Abcam assay, and 90 pg/ml and 172 pg/ml for the Proteintech assay. For the GeneTex assay, LOD was > 200 pg/mL, so the LOQ was not calculated in more detail. The Roche-Elecsys and EU SARS-CoV-2 antigen tests had a good correlation (R^2^ = 1) (data not shown)

A highly concentrated SARS-CoV-2 EQA sample distributed in March 2021, EQA Scheme Prog. 410 (EQA sample no. 410,004), was used to detect and measure the NCap by using different ELISA tests. NCap quantification was significantly lower when using the GeneTex assay (965 pg/ml) as compared to Abcam (2509 pg/ml) and Proteintech (2213 pg/ml). To verify the accuracy of the results from the samples investigated, we assessed the linearity at different levels of dilution in the diluent buffer of each ELISA kit (Fig. 1). Of the quantitative tests investigated, the Abcam test achieved the best linearity over the different dilutions (Fig. 1).

**Fig. 2:**
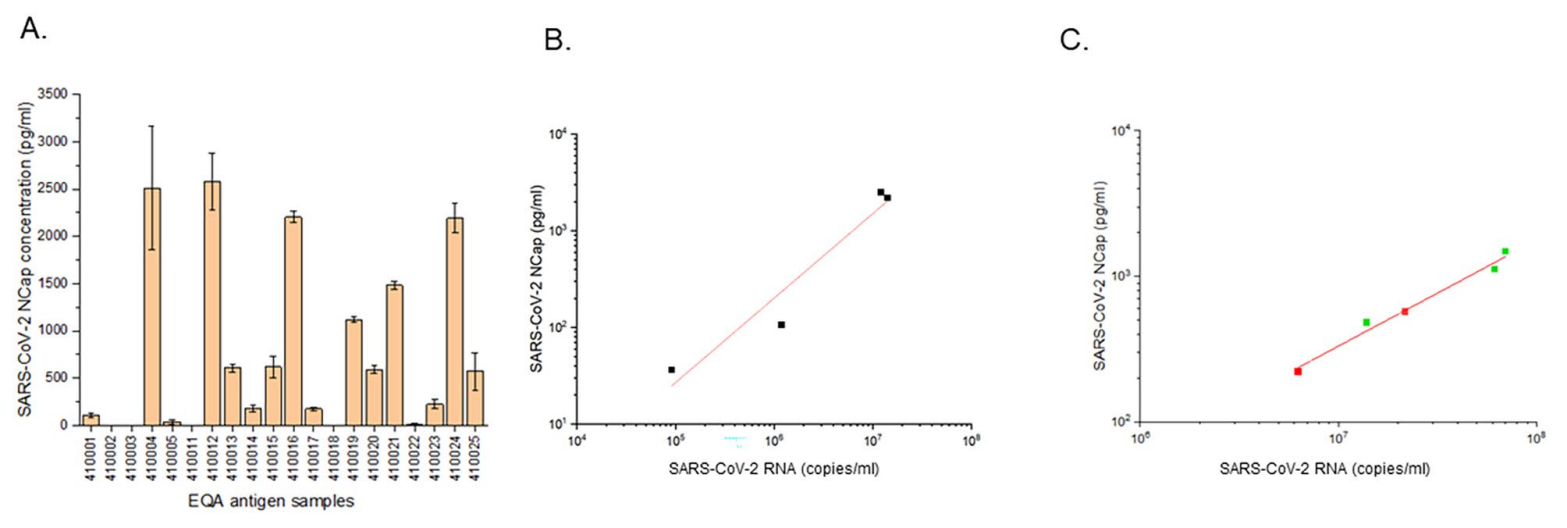
**A.** SARS-CoV-2 nucleocapsid quantification of EQA samples from Table 1 (mean ± SD); **B.** Correlation analysis of nucleocapsid with RNA concentration using different dilutions of SARS-CoV-2 Non-VOC, BetaCoV/Munich/ChVir984/2020_IsolatBER (ID. 410,001 410,004, 410,005 and 410,024 (black dots), R^2^ = 0.93. Intercept: -2.94 ± 1.04 (pg/ml); Slope: 0.87 ± 0.16 (pg/copies); **C.** Correlation of nucleocapsid with RNA concentration using different dilutions of two VOC SARS-CoV-2 variants: SARS-CoV-2 Delta VOC, B.1.617.2, hCoV-19/Germany/SH-CHVir25702_4/2021 (EQA 410,015, 410,019 and 410,021) (green dots) and Omicron VOC, hCoV-19/Germany/SH-ChVir26373/2021, Accession ID: EPI_ISL_7495250 (EQA410023 and 410,025) (red dots). R^2^ = 0.98. Intercept: -2.56 ± 0.43 (pg/ml); Slope: 0.73 ± 0.06 (pg/copies). NOTE: Fig. 2B and C have different scales in the “x“ axis

To further validate the Abcam ELISA performance, we analyzed the EQA SARS-CoV-2 samples included in Prog. 410 of INSTAND EQA from September/November 2021 and March 2022 (Table 1). The results were in good agreement with the qualitative target values for each of the EQA samples (Fig. 2A; Table 1).

We also investigated the correlation between SARS-CoV-2 genome and NCap antigen concentration levels using different dilutions of EQA samples of SARS-CoV-2. The NCap antigen concentrations were in the range of 36.33pg/ml to 2509.52 pg/ml for samples with the non-variant of concern (non-VOC) isolate and between 220.75 pg/ml and 1481.05 pg/ml for the samples containing VOCs. The RNA concentrations ranged from 9.1 × 10^4^ copies/ml to 1.2 × 10^7^ copies/ml for the samples containing non-VOC isolates and from 6.2 × 10^6^ copies/ml to 7 × 10^7^ copies/ml for VOC isolates. We demonstrated that both the SARS-CoV-2 non-VOC and VOC showed a good correlation between viral RNA and NCap antigen, with R^2^ = 0.93 and 0.98, respectively (Fig. 2B C). However, this correlation is not one-to-one (genome-to-protein) and depends on the SARS-CoV-2 isolate tested. For example, the amount of SARS-CoV-2 NCap antigen corresponding to approximately 10^7^ RNA copies/ml was four times higher in the non-VOC isolate (410,004) than in the delta isolate (410,015) (Table 1).

## Discussion

The direct correlation of RNA and protein concentrations in SARS-CoV-2 as measured by diagnostics tests is still poorly understood. To date, RT-qPCR is considered the gold standard for SARS-CoV-2 detection [12]. But this routinely used test has limitations regarding the quantification of the viral load of SARS-CoV-2 [13, 14]. By contrast, RT-dPCR is a calibration-free quantitative method that demonstrates better reproducibility and sensitivity than RT-qPCR [10, 15]. This work is the first characterization of SARS-CoV-2 NCap antigen concentration using highly stable and homogeneous samples from an EQA scheme (INSTAND) and correlating the results with RT-dPCR measurements. To correlate SARS-CoV-2 NCap and RNA concentration, we first chose the quantitative ELISA kit with the best performance in terms of linearity, LOD and LOQ. Our results suggest that the Abcam kit shows the best dilution linearity over the different dilutions of the EQA sample (R^2^ = 0.999) as well as the lowest LOD and LOQ. As such, it is the preferred assay for quantifying NCap antigen. The performance of the Proteintech kit is in terms of dilution linearity and LOD/LOQ less satisfactory than that of the Abcam kit. Moreover, this study indicates that GeneTEX protein quantification is less efficient and accurate than the other quantitative ELISA kits used here, showing poor dilution linearity and very high LOD and LOQ for protein measurements. Therefore, GeneTEX does not appear to be useful for NCap quantification in EQA samples.

In principle, if we compare the results of RT-dPCR and ELISA measurements using the dilution series of EQA samples, we expect to find a perfect one-to-one correlation. However, our study indicates no such one-to-one correlation of SARS-CoV-2 RNA and NCap antigen among the dilution series of the studied EQA samples. Hence, the amount of SARS-CoV-2 NCap antigen cannot be predicted based on the samples’ genome concentration. There are many factors affecting the correlation, including the nature of the SARS-CoV-2 isolate.

## Conclusions

In conclusion, our data suggest that constant evaluation of the sensitivity of ELISA for detecting new SARS-CoV-2 variants is vital to support monitoring and antigen testing. Further analysis including clinical samples and RT-dPCR absolute quantification will also help us to better understand the correlation between RNA and NCap antigen.

### Limitations

The limitation of this study is the reduce number of SARS-CoV-2 variants of concern and non-variants of concern samples used; additionally clinical samples should be also tested to better understand the correlation between RNA and NCap antigen.

## Data Availability

All data generated or analysed during this study are included in this article.

## Abbreviations

COVID-19: Coronavirus disease 2019
EQA: External quality asurance
LOD: Limit of detection
LOQ: Limit of quantification
NCap: Nucleocapsid
Non-VOC: Non-variant of concern
RT-dPCR: Reverse transcriptase digital polymerase chain reaction
RT-qPCR: Reverse transcriptase quantitative polymerase chain reaction
SARS-CoV-2: Severe acute respiratory syndrome coronavirus 2
VOC: Variant of concern
